# Application of small RNA technology for improved control of parasitic helminths

**DOI:** 10.1016/j.vetpar.2015.06.003

**Published:** 2015-08-15

**Authors:** Collette Britton, Alan D. Winter, Neil D. Marks, Henry Gu, Tom N. McNeilly, Victoria Gillan, Eileen Devaney

**Affiliations:** a Institute of Biodiversity, Animal Health and Comparative Medicine, University of Glasgow, Bearsden Road, Glasgow G61 1QH, UK; b Moredun Research Institute, Pentlands Science Park, Bush Loan, Penicuik EH26 0PZ, UK

**Keywords:** Nematode, Helminth, MicroRNA, RNA interference, Gene regulation, Diagnosis

## Abstract

Over the last decade microRNAs (miRNAs) and small interfering RNAs (siRNAs) have emerged as important regulators of post-transcriptional gene expression. miRNAs are short, non-coding RNAs that regulate a variety of processes including cancer, organ development and immune function. This class of small RNAs bind with partial complementarity to their target mRNA sequences, most often in the 3′UTR, to negatively regulate gene expression. In parasitic helminths, miRNAs are being increasingly studied for their potential roles in development and host-parasite interactions. The availability of genome data, combined with small RNA sequencing, has paved the way to profile miRNAs expressed at particular developmental stages for many parasitic helminths. While some miRNAs are conserved across species, others appear to be unique to specific parasites, suggesting important roles in adaptation and survival in the host environment. Some miRNAs are released from parasites, in exosomes or in protein complexes, and the potential effects of these on host immune function are being increasingly studied. In addition, release of miRNAs from schistosome and filarial parasites into host plasma can be exploited for the development of specific and sensitive diagnostic biomarkers of infection. Interfering with miRNA function, as well as silencing key components of the pathways they regulate, will progress our understanding of parasite development and provide a novel approach to therapeutic control. RNA interference (RNAi) by siRNAs has proven to be inconsistent in parasitic nematodes. However, the recent successes reported for schistosome and liver fluke RNAi, encourage further efforts to enhance delivery of RNA and improve *in vitro* culture systems and assays to monitor phenotypic effects in nematodes. These improvements are important for the establishment of reliable functional genomic platforms for novel drug and vaccine development. In this review we focus on the important roles of miRNAs and siRNAs in post-transcriptional gene regulation in veterinary parasitic helminths and the potential value of these in parasite diagnosis and control.

## Highlights

• MicroRNAs and siRNAs in helminth post-transcriptional gene regulation are reviewed.
• Many parasitic helminth miRNAs are unique and developmentally expressed.
• miRNAs released by parasites have diagnostic potential, particularly for filarial and schistosome spp.
• Parasite and host miRNAs may regulate immune responses.
• Improvements to siRNA-mediated gene silencing are important for functional genomics.

## Graphical abstract

**Figure:**
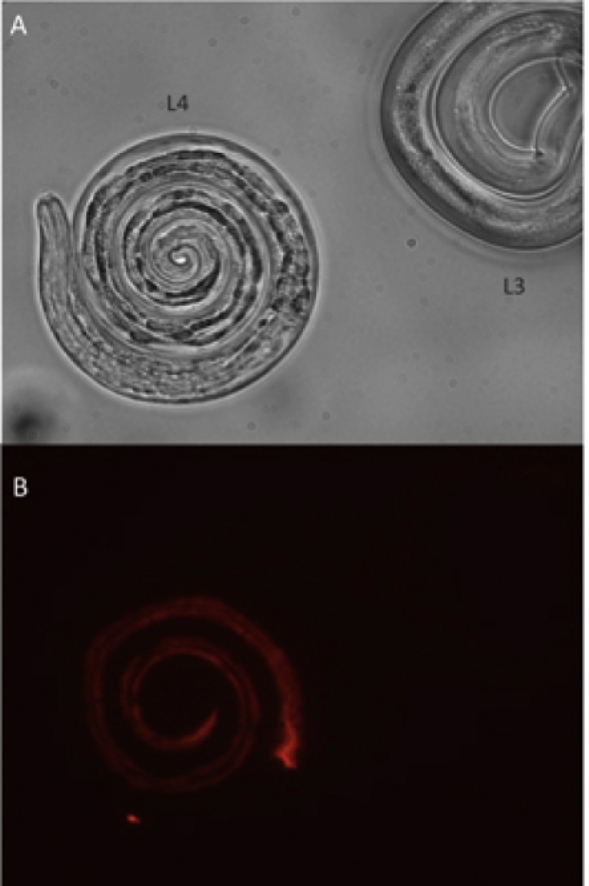

## 1 Introduction

Parasitic helminths are responsible for more than 55% of all of livestock diseases in Europe and cause significant productivity losses and welfare problems globally (Morgan et al., 2013). Economic losses due to infection of cattle and sheep with the liver fluke *Fasciola hepatica* are estimated at US $3 billion per annum globally (Piedrafita et al., 2010). In the UK alone, gastrointestinal (GI) nematode infections are conservatively estimated to cost the sheep industry £84 million per annum in production losses and treatments (Nieuwhof and Bishop, 2005). Control of nematode and fluke infections currently relies on the administration of anthelmintic drugs. However, their widespread use has resulted in development of resistance to the commonly used drug classes (benzimidazoles, imidazothiazoles such as levamisole and macrocyclic lactones including ivermectin). Drug resistance is a major concern to sheep farming, particularly in Australia, New Zealand and Brazil, where multi-drug resistance is widespread. Resistance is also being increasingly reported in cattle parasites (Sutherland and Leathwick, 2011), with significant economic implications. Alternative methods of control including improved management, vaccination and new drug development are urgently needed. A better understanding of parasite biology is important for the identification of novel targets of control, and the sequencing of parasite genomes over the last few years provides a powerful resource for new drug and vaccine discovery. In addition, transcriptome data presents a changing picture of gene expression as parasites develop and can identify genes enriched in particular tissues. Genome and transcriptome data are now available for a range of helminths. (http://parasite.wormbase.org/info/website/species.html) including the small ruminant GI nematode *Haemonchus contortus* (Laing et al., 2013; Schwarz et al., 2013), the pig nematodes *Ascaris suum* and *Trichuris suis* (Jex et al., 2011, 2014), human hookworm *Necator americanus* (Tang et al., 2014) filarial (Ghedin et al., 2007; Godel et al., 2012) and schistosome species (Berriman et al., 2009; The Schistosoma japonicum Genome Sequencing and Functional Analysis Consortium, 2009) and liver fluke *F. hepatica* (Cwiklinski et al., 2015). The availability of sequence data is a major progression; exploiting these large datasets to learn more about how parasites develop and survive within the host and identify how we may interfere with these processes is the obvious next step.

Over the last decade, there has been increasing interest in small RNAs in parasitic nematodes. Firstly, small interfering (si) RNAs, generated from double-stranded RNA (dsRNA), can act to silence gene expression and have potential as a tool to identify gene function. RNA interference (RNAi)-mediated gene silencing has been applied to a range of parasitic helminths, with most success currently reported for *Schistosoma* species (Bhardwaj et al., 2011). The second class of small RNAs, microRNAs (miRNAs), have been identified as important regulators of nematode development and are also released from parasites into the host environment. This has led to significant interest in miRNAs as mediators of host-parasite interactions and as diagnostic markers of infection. A third class of small RNAs, piwi-interacting (piRNAs) are involved in silencing transposons in the germ-line and are restricted to clade V parasitic nematodes (Winter et al., 2012; Sarkies et al., 2015); other nematode clades appear to have adopted different mechanisms for this same function (Wang et al., 2011; Sarkies et al., 2015). This review will focus on miRNAs and siRNAs in veterinary parasitic helminths and their important functions in the post-transcriptional regulation of gene expression. We will also discuss applications of small RNA technology to parasite diagnosis and as a potential novel approach to parasite control.

## 2 MicroRNAs

miRNAs are non-coding RNAs of 21–25 nucleotides in length. They are processed from long primary transcripts through the action of Drosha ribonuclease to form pre-miRNAs with stem-loop structures of around 70 nucleotides (Kim et al., 2009). Cleavage by Dicer results in duplex RNAs of around 22 bp, one strand of which is incorporated into the RNA-induced silencing complex (RISC) (Chekulaeva and Filipowicz, 2009). miRNAs regulate gene expression post-transcriptionally by binding with partial sequence complementarity, most commonly to the 3′ untranslated region (UTR) of their target mRNAs. Each miRNA can target many mRNAs and each mRNA sequence can be targeted by many miRNAs, adding an additional layer of complexity to gene regulation. miRNAs direct the RISC to the target sequence, resulting in translational repression and/or mRNA destabilisation. Nucleotides 2–8 of the mature miRNA, referred to as the seed sequence, are the most important in determining binding specificity, although binding energies at other parts of the miRNA-mRNA interaction are a contributing factor.

The importance of miRNAs was first demonstrated in the free-living nematode *Caenorhabditis elegans*. *C. elegans lin-4 and let-7* were identified as non-coding RNAs essential for correct developmental progression from larval stages L2 to L3 and from L4 to adult worms, respectively (Lee et al., 1993; Reinhart et al., 2000). Both miRNAs negatively regulate expression of genes involved in hypodermal cell fate. In *C. elegans, lin-4* or *let-7* loss of function leads to phenotypes opposite to that of target gene loss, demonstrating the inverse nature of this regulation. The identification of *let-7* in higher organisms, in which the sequence is perfectly conserved, resulted in the recognition of the widespread importance of miRNA-mediated gene regulation. In mammals, miRNAs regulate a range of diverse and important processes including insulin secretion (Poy et al., 2004), cardiogenesis (Zhao et al., 2005) and cancer development (He et al., 2007). In livestock, a number of economically important traits, including muscle growth, lactation and fertility, are associated with breed-dependent variation in expression of specific miRNAs (Fatima and Morris, 2013), highlighting the potential application of miRNAs in selective breeding programmes.

### 2.1 microRNAs as potential novel therapeutics for parasite control

Analysis of *C. elegans* strains with mutations in individual miRNAs or miRNA families has demonstrated essential functions for some of these. For example, *C. elegans lin-4* and *let-7* family members are important for correct larval development, the *mir-36* family is essential for embryonic development, the *mir-51* family is required for pharynx attachment during embryogenesis and the *mir-58* family is involved in regulating locomotion, growth and development of arrested dauer stage larvae (Alvarez-Saavedra and Horvitz, 2010).

The availability of genome sequence paved the way for characterisation of miRNAs in parasitic helminths. Initial studies employed bioinformatic approaches (BLASTN, RNAfold, MapMi) (Guerra-Assuncao and Enright, 2010) to identify conserved miRNAs and their precursor stem-loop structures. Subsequent sequencing of small RNA libraries has allowed identification of many novel parasite miRNAs. Detailed information on miRNAs expressed by parasitic helminths has been generated through analysis of small RNA libraries from different developmental stages and/or tissues and probing of miRNA microarrays. These approaches have been used to characterise miRNA expression in a range of nematodes, fluke and tapeworms (reviewed in Britton et al., 2014) including *A. suum* (Wang et al., 2011), *H. contortus* (Winter et al., 2012), *Brugia* species (Winter et al., 2012; Poole et al., 2014), *Trichinella spiralis* (Chen et al., 2011a), *Angiostrongylus cantonesis* (Chen et al., 2011b), *Taenia saginata* (Ai et al., 2012), *Echinococcus* (Cucher et al., 2011, 2015) and schistosomes (Huang et al., 2009; Simoes et al., 2011). miRNA sequences from parasitic helminths and many other organisms are available from miRBase (http://www.mirbase.org). While some miRNAs are conserved, suggesting important functions, many are unique to each species, at least based on current data. This suggests that miRNAs are rapidly evolving and may be important in parasite transmission and adaptation to different environments. However, as data is generated from more parasites, it is likely to reveal conservation of miRNAs in related species, occupying similar host niches. Indeed, recent small RNA sequence data we have generated from the ovine GI nematode *Teladorsagia circumcincta* shows strong homology to *H. contortus* miRNA sequences, indicating conservation of miRNAs between related trichostrongylids.

Detailed analysis of miRNAs expressed in *H. contortus* has identified a significant number that are unique to *H. contortus* or unique to nematodes and not found in higher organisms. Expression of many of these is temporally regulated in post-infective stages, suggesting important roles in parasite development and interaction with the host environment (Winter et al., 2012). These miRNAs and the genes and pathways they regulate are therefore of interest as potential novel targets of control. Identifying the functions of specific miRNAs in parasitic species, to select those of most relevance as control targets, is not an easy task. The increasing availability of well-annotated genome data for parasitic helminths and generation of 3′UTR datasets is significantly advancing the bioinformatic prediction of target mRNAs. For some helminth species, genome annotation is still in progress, thus limiting the application of UTR analysis tools. However, as assembly and annotation improve for more helminth genomes, this should soon be feasible on a wider scale. A number of target prediction algorithims can be applied to score miRNA binding sites in input sequences (reviewed in Thomas et al., 2010). Complementing this is the increasing transcriptome data; the inverse correlation between miRNA and messenger RNA (mRNA) target gene expression can help filter potential target genes, although some miRNAs may have only a modest effect on mRNA levels. We recently applied bioinformatics approaches to identify predicted targets of a novel microRNA (miR-5364), which is significantly upregulated in *Brugia* larvae following infection of the mammalian host (Winter et al., 2015). The target gene set was refined using comparative genomics and transcriptomic analysis. A luciferase reporter assay in transfected HEK293 cells was used to experimentally confirm the interaction between *mir-5364* and predicted target gene 3′UTRs. Biochemical pull-down approaches can also be applied to identify miRNA target genes present in the RISC. This approach, referred to as HITS-CLIP (high throughput sequencing by cross-linking immunoprecipitation), has been used successfully in *C. elegans* (Zisoulis et al., 2010) and we are currently adapting this methodology for *H*. *contortus* target identification.

**Fig. 1:**
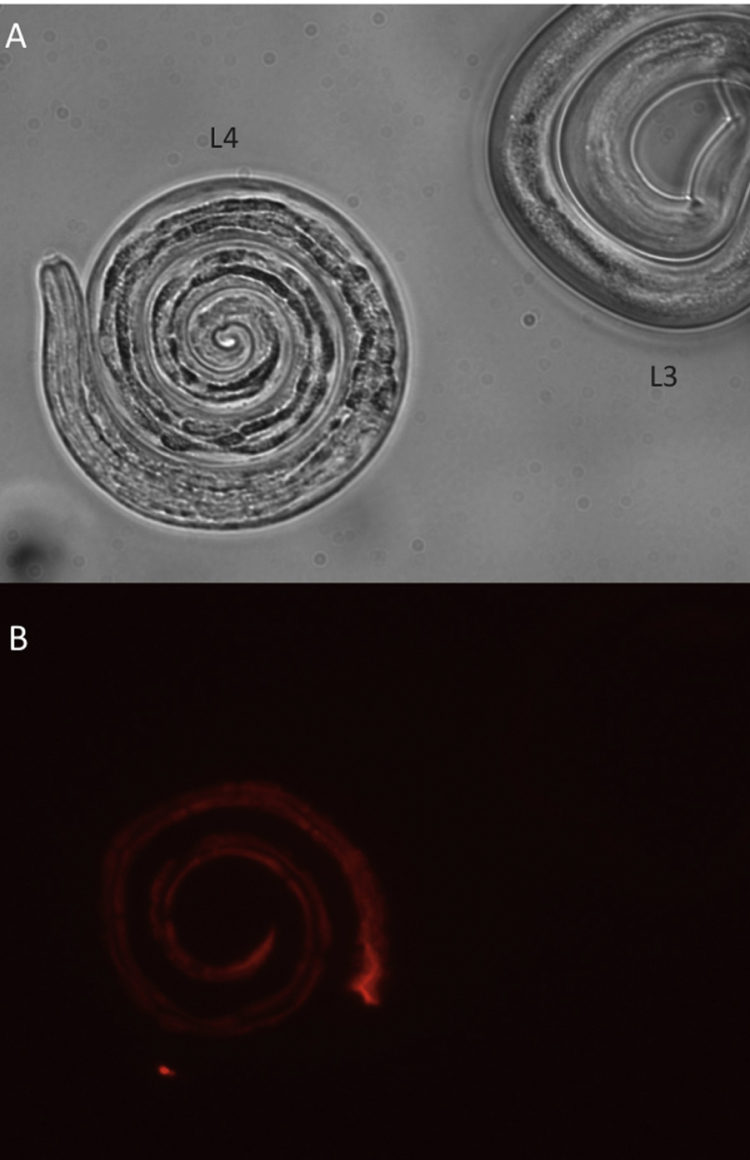
Uptake of fluorescently-labelled siRNA by *Haemonchus contortus* L4 larvae *in vitro.* 1000 L3 larvae were exsheathed and incubated in EBSS/5% CO_2_ at 37 °C in the presence of 0.5 μM Cy3-labelled siRNA (Ambion). After 96 h, a sample containing larvae at L3 and L4 developmental stages was washed three times in PBS and pipetted onto a 2% agarose pad on microscope slides and viewed by bright field (A) or fluorescent (B) microscopy at ×40 magnification. Images were collected using an Axioskop 2 Plus microscope (Zeiss), ORCA-ER digital camera (Hamamatsu) and Openlab (Improvision) software. Fluorescence is observed throughout the intestine of the L4. No fluorescence was observed in the L3 stage or in any stage in the absence of Cy3-labelled siRNA. Larvae are approximately 700 μM in length.

As a result of the importance of miRNAs in human diseases including cancer, the chemistry involved in the design of stable miRNA inhibitors and mimics is well-defined. Indeed several miRNA inhibitors and mimics are undergoing clinical trials, the most notable being a miR-122 inhibitor (miravirsen) for control of hepatitis C virus infection (Janssen et al., 2013). miRNA inhibitors are anti-sense oligonucleotides designed to the mature miRNA and can be modified to withstand endogenous nucleases and aid cellular uptake (reviewed in Van Rooij et al., 2012). For example, incorporation of locked nucleic acids (LNA) containing bridged oxygen and carbon ribose molecules, 2′-*O*-methyl group modifications and phosphorothioate bonds all enhance stability. We have demonstrated significant uptake of fluorescently-labelled small RNAs by larval and adult stages of *B. pahangi* (Winter et al., 2015) and L4 stage of *H. contortus* developed *in vitro* (Fig. 1). We are currently testing the effects of miRNA inhibitors and mimics of specific miRNAs that are significantly up or down regulated in the transition of *H. contortus* to the post-infective L4 stage. Although uptake of small RNAs is demonstrable, it is possible that improvements to *in vitro* culture systems will be needed for phenotypic detection, as discussed below for RNAi. In addition, as miRNAs can act together to target particular genes and pathways, inhibition of co-expressed or related miRNAs may be necessary (discussed in Winter et al., 2015).

### 2.2 Diagnostic application of parasite microRNAs

Changes in miRNA expression during human pathologies, including cancer and tissue damage, can be detected in blood, plasma and urine samples. Much attention is therefore focussed on the potential of miRNAs as specific disease biomarkers (Brase et al., 2010). For parasitic helminth infections, a number of recent studies have identified parasite miRNAs in serum or plasma, specifically from infected hosts. Whether these are released from live or dying parasites is not known. However the extreme stability of miRNAs in serum, combined with the high sensitivity and specificity of detection, makes miRNAs attractive as potential biomarkers of parasitic infection, particularly where current diagnosis is difficult. Sequencing of small RNAs from the serum of mice infected with *S. mansoni* identified both host and parasite miRNAs (Hoy et al., 2014). Importantly, three of the parasite-derived miRNAs (bantam, miR-277 and miR-3479-3p) were also detected by qRT-PCR in serum from humans infected with *S. mansoni.* It was possible to distinguish egg-positive from egg-negative individuals, demonstrating the relevance of these miRNAs as biomarkers. For the dog heartworm, *Dirofilaria immitis,* over 200 potential parasite-derived miRNAs were identified by sequencing of small RNAs present in plasma from infected dogs (Tritten et al., 2014). Five of the most abundant miRNAs, based on sequence read number, were selected for qRT-PCR amplification and two of these (miR-71 and miR-34) were readily amplified using plasma from infected but not from uninfected dogs. This study demonstrated the specificity of miRNA detection, although there was no correlation between miRNA copy number and host microfilariae counts (Tritten et al., 2014).

Conservation of miRNA secretion by filarial nematodes was shown recently in a study by Quintana et al. (2015). Small RNA analysis of nodule fluid from cattle infected with *Onchocerca ochengi* identified 62 miRNAs, 59 of which were identical to miRNAs in the closely related human parasite *O. volvulus*. Six of the miRNAs found in *O. ochengi*-infected nodule fluid were also detected in serum from *O. volvulus* infected humans and four were present in serum of mice infected with *Litomosoides sigmodontis*. A common theme is the release of miR-71*,* miR-100 family members and bantam from filarial species into the host circulation; a distinct bantam sequence is also present in serum of humans and mice infected with *S. mansoni*. The accumulating data indicate the utility of specific parasite miRNAs released into the circulation as potential diagnostic markers of infection.

Perhaps not surprisingly, given the restriction of the parasite to the GI tract, no parasite-derived miRNAs were detected in the serum of mice infected with the nematode *Heligmosomoides polygyrus* (Buck et al., 2014). We also failed to detect known abundantly-expressed miRNAs of *H. contortus* in serum from infected sheep. However, we were able to detect *H. contortus*-derived miRNAs by small RNA library sequencing and/or qRT-PCR in *H. contortus* adult worm excretory-secretory (ES) products and in the draining lymph node and abomasal tissue from infected but not from uninfected sheep. This observation suggests that gut-dwelling nematodes may release miRNAs into the local gut environment, where they can act at both the mucosa as well as associated mucosal lymphoid tissue. Parasitic helminth infections are characterised by modulation of host immune responses that benefit parasite survival. Whilst most research in this area has focussed on immunosuppressive activities of nematode ES products or specific secreted proteins (Hewitson et al., 2009; McNeilly et al., 2013), miRNAs may also have important influence on immune outcome. Elegant studies on *H. polygyrus* ES material have demonstrated that some secreted miRNAs are packaged within exosomes (extracellular vesicles) that likely originate from the nematode intestine (Buck et al., 2014). Interestingly, administration of these vesicles to mice suppressed the induction of a Th2-like innate response following allergen exposure. Whether this effect is mediated specifically by miRNAs is not yet known but it has been shown that miRNAs within exosomes are delivered to host cells *in vitro* (Buck et al., 2014). Release of exosomes derived from the tegument of the liver fluke *F. hepatica* has also been demonstrated (Marcilla et al., 2012). Although miRNAs were not investigated in the study, these fluke exosomes were found to contain immunomodulatory molecules, including cathepsin L, peroxiredoxins and helminth defense molecule (HDM). These were also described in fluke ES products, and may explain the lack of typical secretory signal sequences on fluke proteins. Importantly, miRNAs as well as proteins were identified in exosomes of the trematode *Dicrocoelium dendriticum* (Bernal et al., 2014). Three of these were previously described in serum from *S. mansoni*-infected humans (see above; bantam, miR-277 and miR-3479). Therefore, in addition to application in helminth diagnosis, further study of the origin and functions of miRNAs released by parasites will be important in advancing our understanding of host-parasite interactions and mechanisms of immunomodulation.

### 2.3 Host miRNA expression during helminth infections

Changes in host miRNA expression following parasitic helminth infections are relevant to our understanding of tissue pathology and immune outcome. One host miRNA (miR-223) has recently been proposed as a biomarker of liver pathology during *S. japonicum* infection (He et al., 2013). In a mouse model, levels of miR-223 increased during infection and, importantly, returned to near normal levels following praziquantel treatment, indicating specific association with infection and not other causes of liver damage. Detection of specific host miRNAs therefore has potential application in monitoring the pathology and progression of parasite infections.

In addition, changes in host miRNA expression during infection may identify novel mechanisms regulating induction of protective or suppressive immune responses and variation between individual hosts. miRNAs have emerged in recent years as important regulators of both innate and adaptive immune responses in a variety of murine model systems (Gantier, 2010). Changes in host miRNAs following parasitic helminth infection have not been extensively studied in veterinary species. However there is good reason to believe that miRNA profiles will be altered following infection with metazoan pathogens and will help determine the outcome of an immune response. Whether these changes arise through modulation of the immune response by the parasite or as a normal component of the mammalian immune response to infection remains unclear. Certainly, infection with viruses and protozoan parasites has been shown to alter host miRNAs resulting in enhanced replication and survival (Skalsky and Cullen, 2010; Marsolier et al., 2013).

A hallmark of many helminth infections is the expansion of alternatively activated macrophages and, using a mouse model of filarial infection, Rückerl et al. (2012) demonstrated that a small number of host miRNAs were differentially expressed when macrophages were elicited to undergo alternative activation. This cell type is also expanded in cattle infected with *F. hepatica* (Flynn et al., 2007) but whether miRNAs are involved in driving this expansion remains to be studied. In a well-characterised mouse model of *S. mansoni* infection, in which regulatory T cells (Tregs) play an important role in restricting pathology, Kelada et al. (2013) identified host miRNAs that are specifically induced during a Th2 response. miR-182 appeared to regulate the expression of a number of genes that are important for the expansion and stability of Tregs under Th2 inducing conditions. Testing the effects of specific parasite molecules on host miRNA expression may provide a novel route to understanding mechanisms of protection and immunomodulation.

## 3 Small interfering RNAs (siRNAs)

A separate class of RNAs, referred to as small interfering (si) RNAs, is also important in post-transcriptional gene regulation. siRNAs are processed from endogenous or exogenous long double-stranded (ds) RNA by Dicer ribonuclease. In *C. elegans,* the primary siRNAs interact with the Argonaute protein RDE-1 to identify and cleave homologous mRNA sequence. Amplification of this response is mediated by secondary siRNAs produced by RNA-directed RNA polymerases RRF-1 or EGO-1 (Grishok, 2005; Zhuang and Hunter, 2012). In contrast to miRNAs, which are often derived from intergenic or intronic genomic locations, siRNAs map anti-sense to coding sequences. siRNAs are the key mediators of RNA interference-mediated gene silencing (RNAi), a pathway that is thought to have evolved to control transposons and dsRNA viral infection. Indeed, *C. elegans* isolates or mutants defective in RNAi show greater susceptibility to infection with Orsay virus, a natural virus of *C. elegans* (Felix et al., 2011). No natural viruses have yet been identified in parasitic helminths to determine whether RNAi plays a similar defensive role in these species. A number of studies have reported RNAi gene silencing in a range of parasitic helminths, supporting the presence and activation of a functional silencing pathway. Exploiting this mechanism to silence genes of interest would provide a much-needed functional genomics tool. This is particularly timely with the increasing availability of helminth genome and transcriptome data.

### 3.1 Gene silencing by RNAi

RNAi has been attempted in a range of parasitic nematodes with the aim of identifying essential gene function. While knockdown of target mRNA level is possible, results have often been inconsistent (reviewed in Britton et al., 2012; Dalzell et al., 2011) with variations between different dsRNA preparations and batches of larvae. A recent RNAi study in *T. circumcincta* showed an age-dependent effect, with L3 infective larvae stored for more than two months at 4 °C being refractory to RNAi following soaking in dsRNA (Tzelos et al., 2015). Most studies have demonstrated significant silencing of some target genes, but not others. By selecting target genes based on site and level of expression, our findings suggest that mRNAs expressed in sites accessible to dsRNA (intestine, excretory/secretory cell, amphids) are more susceptible to silencing via soaking (Samarasinghe et al., 2011). While this approach may improve the success of RNAi, current methods of gene knockdown are limited and not amenable to genome-wide analysis.

Improvements to RNA delivery and *in vitro* culture systems to enable phenotypic detection are needed. For example, we demonstrated effective knockdown of the H11 aminopeptidase vaccine candidate in *H. contortus* L3 larvae following soaking. While no phenotype was observed *in vitro*, infection of sheep with larvae soaked for 24 h in H11 dsRNA resulted in reduced worm burden and egg output compared to control-treated larvae (Samarasinghe et al., 2011). Together these data suggest that current *in vitro* culture methods limit our ability to detect important gene function.

### 3.2 The future for RNAi in vaccine design and drug discovery

With the increasing resistance to widely used anthelmintic drugs, reliable tools are needed to exploit genome and transcriptome data to identify novel drug and/or vaccine candidates. This is particularly important for liver fluke and GI nematodes of sheep and cattle where drug resistance is an increasing problem. Application of RNAi for the identification of control targets will require more reliable methods of silencing. Recent studies in the liver fluke *F. hepatica* have shown robust gene silencing; knockdown can be achieved after only four hours of soaking in dsRNA or siRNAs and is stable for at least 25 days (McVeigh et al., 2014). Significant knockdown has been reported by the Maule lab for a range of genes including neuronally-expressed genes, which in *C. elegans* are refractory to RNAi. As several classes of current anthelmintic drugs target neuronal receptors, effective silencing of neuronal genes is important to future drug discovery.

Silencing of neuronal genes by RNAi is also effective in schistosome parasites. Recent studies have demonstrated effects on motility of schistosomula and adult worms following RNAi of G protein-coupled receptor and nicotinic acetylcholine receptor genes (MacDonald et al., 2014; Patocka et al., 2014). With the availability of genome data for *Schistosoma* spp. and the increasing number of reports of successful silencing, RNAi is being applied more widely to identify potential new control targets. Detailed studies in *F. hepatica* suggest that the slow rate of protein turnover, at least *in vitro*, is a current hurdle in identifying essential gene function in this parasite. Transcript knockdown is rapid and sustained, as demonstrated by qRT-PCR, but there is a significant lag time before protein levels are reduced (>21 days for some targets). This most likely explains the lack of phenotypes observed (McVeigh et al., 2014). Efforts to improve functional assays and *in vitro* culture systems employing complex medium or cell co-culture, as well as *in vivo* studies will be important in progressing application of RNAi as a functional genomics platform. Development of high throughout screening approaches would be particularly advantageous, such as soaking in dsRNA, siRNA or feeding on bacterially-expressed dsRNA.

## 4 Conclusion

The availability of genome data for parasitic helminths has paved the way for identification of miRNAs involved in gene regulation and stimulated efforts in applying siRNA-mediated gene silencing. Secretion of specific miRNAs from parasitic helminths can be exploited to develop sensitive and novel diagnostic markers of infection. Moreover, identifying the roles of miRNAs released by parasites and following changes in host miRNA expression during infection will advance our understanding of host-parasite interactions. An improved understanding of the miRNAs that regulate key target genes and pathways has potential to lead to novel approaches for control. These studies will be complemented by ongoing efforts to improve gene silencing in helminth parasites. The successes achieved with RNAi in parasitic and free-living planarian flatworms are highly encouraging and suggest that the development of functional tools for nematodes should be feasible.
